# Characteristics and challenges of the clinical pipeline of digital therapeutics

**DOI:** 10.1038/s41746-020-00370-8

**Published:** 2020-12-11

**Authors:** Nisarg A. Patel, Atul J. Butte

**Affiliations:** 1 Department of Oral and Maxillofacial Surgery, University of California San Francisco, San Francisco, CA USA; 2 Bakar Computational Health Sciences Institute, University of California San Francisco, San Francisco, CA USA

**Keywords:** Therapeutics, Drug regulation

## Abstract

In this Comment, we characterize the current pipeline of digital therapeutics and offer a clinical perspective into the advantages, challenges, and barriers to implementation of this treatment modality for patient care, which we hope will inform future regulatory policy, prescribing decisions, and scope of real-world evidence collection.

Digital therapeutics (DTx), defined by the Digital Therapeutics Alliance as “evidence-based therapeutic interventions driven by high-quality software programs to prevent, manage, or treat a medical disorder or disease^1^”, have emerged as a new therapeutic modality for the prevention, management, or treatment of chronic, behavior-modifiable disease. Akin to biopharmaceuticals and medical devices, DTx undergo review and are cleared or approved by the U.S. Food and Drug Administration (FDA) and are either available over-the-counter or prescribed by physicians. As of this writing, the FDA has cleared or approved multiple DTx on the basis of superiority trial data, such as Welldoc’s BlueStar for the management of Type II diabetes in 2010 and Pear Therapeutics’ reSET for the treatment of substance use disorder in 2017^2^. For the duration of the COVID-19 public health emergency, the FDA has relaxed regulatory requirements to increase access to digital health products for remote monitoring^3^ and the management of psychiatric conditions^4^, leading to the temporary commercialization of additional DTx under development^5^. Although under consideration, the European Medicines Agency has not yet designed a regulatory pathway for evaluation and commercialization of DTx^6^.

DTx companies have attracted significant venture financing to compete with traditional biotechnology companies developing drugs for similar indications. Pear Therapeutics, a company developing DTx for substance and opioid use disorder, has raised $139 million and Akili Interactive, a company developing DTx for Attention Deficit Hyperactivity Disorder and Autism Spectrum Disorder, has raised $119 million^7^. Yet, a predictable regulatory model to ensure patient safety and the patient, provider, and payer appetite for these therapies remain uncertain.

DTx companies currently target chronic, behavior-modifiable conditions, including Type II diabetes, substance use disorder, autism spectrum disorder, and major depressive disorder. Many of these indications are currently treated with an evidence-based behavioral or psychological mode of management; DTx package these otherwise in-person services into a digital product. For example, reSET serves as a monotherapy to treat patients with alcohol, cannabis, cocaine, or stimulant use disorder by delivering virtual cognitive behavioral therapy (CBT) through a smartphone or tablet. DTx can also act as combination therapy with biopharmaceuticals; for example, reSET-0 provides digital CBT as an adjunct therapy to buprenorphine for patients with opioid use disorder^8^.

If the DTx is prescription-only, physicians must currently complete and send a patient enrollment to the DTx manufacturer, who then onboards the patient directly onto the DTx via a mobile app store access code. Patients interact with the DTx over a predetermined period of time, per the drug’s label, directly from a personal smartphone or tablet; clinicians may also be able to monitor their patients’ progress and input-related information, such as drug screen results and appointment compliance, on a dedicated web dashboard^9^.

DTx may serve as a useful complement, and in certain cases, replacement, to biomedical therapeutics. DTx to date have largely targeted neurological and psychiatric conditions with significant unmet needs that are challenging to manage with existing therapeutics. DTx also allow clinicians to remotely collect real-time data from patients, making subsequent office visits more efficient. Because DTx are software-based, patient progress and adherence to treatment may be more accurately measured relative to pharmaceuticals. Decreased capital requirements and lower risk profile for DTx may also translate into lower prices for DTx relative to novel therapeutics for the same indication. However, how and whether DTx will be evaluated against conventional therapeutics through comparative effectiveness studies also has yet to be explored. Finally, akin to many biotech startups, DTx companies have partnered with large pharmaceutical companies and their investment arms for the development, licensing, and commercialization of their products (Table 1). Understanding and overcoming obstacles to effective regulation and reimbursement of DTx is a key element to the development of long-lasting partnerships with traditional pharmaceutical companies and movement of DTx into the clinic.

**Table 1:** Sampling the Digital Therapeutics Pipeline.

Company	Product	Indication(s)	Status	Commercial partner	Investment partner
Pear Therapeutics	reSET	Substance use disorder^25,26^	Marketed	Sandoz^a^^27^	Novartis
	reSET-O	Opioid use disorder^28^	Marketed	Sandoz^a^	
	Somryst	Chronic insomnia^29^	Marketed		
	Pear-004	Schizophrenia	Marketed^b^ Pivotal		
	Pear-006	Multiple sclerosis	Discovery	Novartis^30^	
	Unspecified	Gastrointestinal conditions	Discovery	Ironwood Pharmaceuticals^31^	
Welldoc	Bluestar	Type I diabetes, Type II diabetes^32^	Marketed	Astellas^33^	
Akili Interactive	Endeavor	ADHD^34^	Marketed	Shionogi^35^	Amgen, Merck
	AKL-T02	Autism spectrum disorder^36^	Pilot		
	AKL-T03	Major depressive disorder	Pilot		
Nightware	Nightware	Post-traumatic stress disorder	Marketed		
Click Therapeutics	CT-152	Major depressive disorder	Pivotal	Otsuka^37^	Sanofi, Hikma
Cognoa	Autism Diagnostic	Autism spectrum disorder	Pivotal	EVERSANA	
	Autism Therapeutic	Autism spectrum disorder^38^	Feasibility		
Biofourmis	BiovitalsHF V1	Heart failure	Marketed	Novartis^39^	
	BiovitalsHF V2	Heart failure^40^	Pivotal		
	BF140	Pain^41^	Pilot	Chugai^42^	
Propeller Health	Propeller	Asthma^43^, chronic obstructive pulmonary disease^44^	Marketed	AstraZeneca^45^, GlaxoSmithKline, Novartis, Orion, Boehringer Ingelheim	
AppliedVR	EaseVRx	Chronic pain^46^	Efficacy		
	RelieVRx	Acute postoperative pain^47^	Efficacy		
	AnxietyVRx	Generalized anxiety	Discovery		
Happify Health	Happify	Multiple sclerosis-associated depression and anxiety	Discovery	Sanofi^48^	

As of November 2020.

^a^Dissolved partnership.

^b^Marketed temporarily under the FDA Enforcement Policy for Digital Health Devices During the Coronavirus Disease 2019 (COVID-19) Public Health Emergency.

## Regulatory considerations

DTx to date have been either granted or cleared by the FDA Center for Devices and Radiological Health following submission of superiority trial data either via the de novo or the 510(k) pathways for medical devices, respectively. Some DTx have also received Breakthrough Therapy designation for expedited regulatory review^10^. However, a dedicated FDA regulatory framework for software-as-a-medical-device (SaMD) products remains in flux^11^. Like traditional pharmaceuticals, some DTx must undergo randomized controlled trials under the premarket approval process to demonstrate safety and efficacy. However, unlike pharmaceuticals, DTx software has the potential to be frequently updated following FDA clearance. Similarly, DTx that rely on artificial intelligence as a component of treatment may function best by “learning” optimal features over time^12^. The FDA regulatory pathway for medical devices, established in 1976^13^, will require modernization to both evaluate and oversee the iterative, autonomous, and adaptive nature of learning SaMD products. In 2017, the FDA announced the Software Precertification Pilot Program, which aims to help “inform the development of a regulatory model to assess the safety and effectiveness of software technologies without inhibiting patient access to these technologies^14^”. Nine companies are currently participating in the pilot program to help the FDA build a Total Product Lifecycle approach to the regulation of software products, and have committed to reviewing real-world performance of their products to ensure patient safety and product quality. In April 2019, to address the challenge posed by adaptive SaMD products, the FDA released a discussion paper describing a possible regulatory approach to premarket review for machine-learning-driven modifications in SaMD^15^. Notably, this document introduces a “predetermined change control plan” to anticipate potential future software updates and a total product lifecycle regulatory approach that aims to facilitate the review of rapid product performance improvements and subsequent deployment without compromising consumer safeguards. In addition, it offers a framework, based on the International Medical Device Regulators Forum risk categorization principles, regarding when a manufacturer would need to submit a new 510(k) for a machine-learning-driven software change to an existing device. The role of academic health systems to run the needed real-world performance studies to help the FDA evaluate and monitor these software products has yet to be defined, and remains a potential growth opportunity.

## Security and data governance

Considerations of cybersecurity and data rights are preconditions for the mass adoption of DTx. Similar to prior work regarding connected sensors in medicine^16^, as DTx transfer information over the internet, risks of unauthorized access and manipulation of these products and underlying data could compromise both trust in the product and patient care. Draft guidance from the FDA regarding cybersecurity in SaMD and networked medical devices was released in October 2018^17^. At this time, medical device manufacturers must comply with federal quality system regulations to address cybersecurity risks of their products both pre- and post-FDA review.

The FDA has defined two “tiers” of devices according to their perceived cybersecurity risk: Tier 1 (higher cybersecurity risk) devices are those capable of connecting, either wired or wirelessly, to another medical or nonmedical product, a network, or the Internet, and for which a cybersecurity breach affecting the device could directly result in patient harm to multiple patients. Tier 2 (standard cybersecurity risk) devices are those for which the criteria for a Tier 1 device are not met. However, while the FDA has issued guidance regarding cybersecurity device design, labeling, and documentation for premarket submissions, the agency does not currently mandate the completion of premarket security audits for medical devices. The current review model relies on device manufacturers to determine the level of cybersecurity risk associated with their product and include a set of cybersecurity design controls to minimize that risk. The FDA then evaluates the manufacturer’s design and risk management documentation for reasonable assurance of safety and effectiveness. However, one study found that from 2002 to 2016, only ~2% of software-enabled device FDA product summaries included cybersecurity content^18^. In 2016, the most recent year in the study sample, 5.5% of product summaries included cybersecurity content. Although information about device cybersecurity may be present in a product’s full FDA dossier, device summaries are some of the primary public-facing documents used by stakeholders to evaluate a product’s safety and effectiveness. This discrepancy raises the concern that patients and clinicians will be unable to make informed decisions about potential product risks. Another study found that hundreds of US medical device recalls were due to software defects, some of which risked patient harm^19^.

As more networked medical devices and SaMD are developed and commercialized and new cybersecurity threats continue to emerge, inclusion of a standardized, public cybersecurity “bill of materials” in premarket applications, as has been called for by some regulators^17^, may help resolve the information gap between manufacturers and purchasers of SaMDs. In addition, security audits for Tier 1 products subject to premarket approval, whether conducted by the medical device manufacturer or a third party, would be a worthwhile inclusion as a required, rather than currently recommended^20^, component of FDA Annual Reports following commercialization. As Annual Reports are not required of devices cleared through the 510(k) pathway, requesting that those product manufacturers submit an independent cybersecurity risk assessment documentation annually may ensure continued device safety. The annual nature of this documentation may help manufacturers account for new technologies that may compromise existing encryption standards and machine learning models, such as quantum computing and adversarial attacks, respectively. Although security audits alone do not guarantee comprehensive security, requesting audit results, and thus ensuring that manufacturers remain vigilant in making design decisions that minimize risk, may reduce the risk of security breaches.

In regard to data rights and governance, manufacturers may employ End User License Agreements, Terms of Service, and Privacy Policies to establish and convey company and user data rights for monitoring, evaluation, and distribution of collected data. The FDA retains oversight for DTx classified as medical devices per Section 3060(a) of the 21st Century Cures Act and may police unauthorized distribution of patient-reported, behavioral, or biometric data. However, because medical device classification by the FDA is based on the intended use of a product rather than the capabilities of the hardware or software itself, determining whether or not a product falls under FDA purview is often ambiguous. This is often the case for many connected sensor technologies in medicine, which instead may fall under the oversight of the Federal Trade Commission, Federal Communications Commission, National Institute of Standards and Technology, and Office of the National Coordinator for Health Information Technology. The obscure nature of oversight responsibility highlights the importance for early engagement with the FDA regarding the regulatory designation of DTx products under development.

## Reimbursement

Despite significant lobbying efforts from DTx manufacturers, the Centers for Medicaid and Medicaid Services (CMS) has not yet issued clear guidance for reimbursement of DTx. Although CMS guidance is typically a regulatory bellwether for commercial payers, CVS and ExpressScripts, the two largest pharmacy benefit managers, launched inaugural digital health formularies in late 2019^21^. Pharmacies and payers typically rely on FDA National Drug Code (NDC) and National Health Related Items Code (NHRIC) numbers for medical device reimbursement within pharmacies. For some state public insurance programs, receiving reimbursement following award of an NDC is commonplace. Early DTx, such as Welldoc’s Bluestar, received these numbers for the purpose of inclusion in drug compendia and reimbursement^22^. However, in 2016 the FDA ruled that SaMD will be ineligible to receive NDC codes after September 2021^23^. Instead, DTx may apply for a unique device identification (UDI) number from the FDA. Consequently, there has been concern from DTx stakeholders that the purchasers of DTx, including pharmacies and payers, are not yet prepared to transition away from use of NHRIC or NDC numbers, which are only 11 digits compared to the UDI’s 14, presenting a bizarre and nontrivial technical challenge. Education regarding the rigor of DTx product development and clinical testing as similar to traditional pharmaceuticals will be crucial in achieving acceptance from payers, PBMs, clinicians, and patients. Given challenges with traditional medication adherence, it will also be important for DTx developers to design products that engage patients over long time periods, particularly for older patients that may be less comfortable with smartphones or tablets. In addition, it is imperative that, moving forward, the data collected by DTx be designed to make in-person visits between patients and clinicians more efficient and actionable rather than burden clinicians with additional paperwork and information. Finally, as DTx offer the added benefit of collecting patient-reported outcomes in real-time, they may be more likely to take on value-based reimbursement arrangements.

## Future directions

Although DTx companies have largely focused on behavioral and chronic conditions to date, some manufacturers have begun transitioning the scope of their products into acute conditions. For example, Click Therapeutics is developing a DTx for Acute Coronary Syndrome^24^. But as more acute scenarios are being contemplated for DTx, it will be even more critical for regulatory agencies to proactively evaluate, label, and issue guidance for these products. CMS should, at the least, develop Healthcare Common Procedural Coding System code(s) for DTx to facilitate payer reimbursement in the absence of dedicated FDA NDC codes. In addition, the FDA should require submission of either manufacturer-directed or external security audits as a component of annual reporting to the agency following commercialization. This additional requirement may help address trust and security concerns in the aftermath of recent significant breaches in both medical and consumer technology software services. Finally, as DTx companies continue to expand indications and reach the clinic, it will be crucial to both educate stakeholders about their clinical value as well as hold their products accountable to the same levels of scientific rigor and oversight that are expected of traditional prescription drugs.
